# Lactic Acid in Tumour Biology

**DOI:** 10.3390/metabo16010075

**Published:** 2026-01-15

**Authors:** Cristina Cruz, Ignasi Barba

**Affiliations:** 1 Faculty of Medicine, Universitat de Vic-Universitat Central de Catalunya (UVic-UCC), 08500 Vic, Spain; 2 Institute for Research and Innovation in Life Sciences and Health in Central Catalonia (IRIS-CC), 08500 Vic, Spain

**Keywords:** cancer, tumour microenvironment, lactic acid, lactate, Warburg effect, immune function

## Abstract

Lactic acid accumulates in the tumour microenvironment (TME) at concentrations reaching up to 40 mM. Initially, lactic acid was considered merely a metabolic by-product of aerobic glycolysis, a phenomenon commonly referred to as the Warburg effect and observed in the majority of tumours. Recent evidence, however, has demonstrated that lactic acid is not merely a waste product; rather, it plays a pivotal role in tumour biology. High plasma lactic acid levels correlate with increased metastatic potential and lower survival rates. Elevated lactic acid levels in the TME have been shown to suppress antitumour immune responses, facilitate both metastasis and cellular senescence, and might modulate gene expression through novel epigenetic mechanisms such as histone lactylation. This review aims to summarize current knowledge on the multifaceted impact of elevated lactic acid in the TME on tumour progression and biology.

## 1. Introduction

Lactic acid (2-hydroxypropanoic acid) derives its common name from the Latin word lac, meaning milk. It was first identified in sour milk by Karl Wilhelm Scheele in 1780. Later, Louis Pasteur demonstrated that lactic acid results from the bacterial fermentation of sugars, while Justus von Liebig confirmed its consistent presence in the muscular tissue of deceased organisms [1].

In healthy individuals, lactic acid is found in the blood at concentrations of approximately 1–2 mM. Given its dissociation constant (pKa) of 3.86, it exists predominantly in its anionic form, *lactate*, at physiological pH. Unless otherwise stated, throughout this review, the terms lactic acid and lactate will be used interchangeably. Most human cells possess the enzymatic machinery to synthesize and efflux lactate into the extracellular milieu space, a process that accounts for the baseline plasma concentration. Lactate production increases under specific conditions, such as limited oxygen availability during muscle exercise [2] or in rapidly proliferating tissues, including those during fetal development [3]. The excess, extracellularly secreted lactate is subsequently processed via hepatic gluconeogenesis in a pathway termed the Cori cycle [4]. Lactate is now recognized as a major circulating carbohydrate fuel and, together with pyruvate, serves as a redox buffer, helping regulate the NADH/NAD ratio [5].

Tumour cells exhibit a distinct metabolic signature [6,7], most notably the conversion of glucose to lactate even under aerobic conditions with functional mitochondria—a phenomenon known as the Warburg effect [8]. This metabolic shift can lead to lactic acid levels of up to 40 mM in the tumour microenvironment (TME) [9], accompanied by decreased pH (acidification). Elevated lactate concentrations in the tumour microenvironment correlate with increased metastatic potential, whereas tumours with lower lactate levels are associated with improved patient survival [9,10]. While common in many cancers, the Warburg effect is not universal. Documented examples include glioblastoma [11], pancreatic cancer [12], breast cancer [13], and cervical cancer [14], among others.

The TME is a complex, heterogeneous milieu comprising immune cells, stromal cells, vasculature, and the extracellular matrix [15]. It actively promotes immune evasion, thereby diminishing the efficacy of immunotherapeutic strategies [16], and can alter the tumour’s responsiveness to chemotherapy and radiotherapy [17]. Within the TME, lactic acid fulfils multiple roles: it is utilized as an energy substrate, operates as a signalling molecule, and holds clinical significance. The central importance of lactic acid in tumour biology has been highlighted in several recent reviews [18,19,20,21,22]. We aim to explore the wide-ranging impacts of lactic acid on tumour biology while focusing on tumour–stroma metabolic interactions linking lactate metabolism with epigenetics and senescence.

## 2. The Origin of Lactic Acid in the Tumour Microenvironment

As mentioned, metabolic dysregulation is a hallmark of cancer [6,7]. The most prevalent manifestation of this dysregulation is the uncoupling of glycolysis from mitochondrial oxidative phosphorylation (OXPHOS), i.e., the Warburg effect [21].

Once taken up by cells, glucose is metabolized through glycolysis to pyruvate, yielding two molecules of adenosine triphosphate (ATP) and two molecules of reducing equivalent NADH. While ATP provides energy for cellular processes, the intracellular accumulation of NADH can inhibit the continuous flux of glycolysis. To sustain glycolytic activity, NADH must be rapidly oxidized back to NAD^+^. This is achieved by the reduction of pyruvate to lactate, a reaction predominantly catalyzed by the enzyme lactate dehydrogenase (LDH), thereby regenerating NAD^+^.

Empirical evidence, such as the observed correlation between lactate dehydrogenase A (LDHA) expression and elevated lactic acid concentration [23], supports the central role of this enzyme in TME lactate accumulation. It is important to note that LDH exists mainly as two isoforms: LDHA, which favours the reduction of pyruvate to lactate, and LDHB, which primarily catalyzes the reverse reaction, the oxidation of lactate back to pyruvate [24]. This bidirectional capacity enables lactate to be re-assimilated and utilized within cellular metabolic pathways.

To maintain glycolytic flux, newly formed lactate must be exported from the cell. This export is mediated by monocarboxylate transporters (MCTs), a family of proton-linked transporters that mediate lactate and pyruvate exchange [25]. Among them, MCT4 serves as the principal lactate exporter, capable of functioning against steep extracellular lactate gradients [26], leading to extracellular concentrations reaching up to 40 mM [27]. MCT1, by contrast, predominantly facilitates lactate uptake into cells [25]. However, both transporters can mediate bidirectional flux, as effective inhibition of lactate export requires the simultaneous blockade of MCT1 and MCT4 [28]. Although the metabolic basis of the Warburg effect involves multiple glycolytic enzymes [8], the net outcome is the accumulation of lactic acid and subsequent acidification of the tumour microenvironment.

Tumour cells represent the primary source of lactic acid within the tumour microenvironment, but stromal components also contribute. For instance, macrophages undergo a metabolic shift from a resting state to a differentiated state that includes the robust production and secretion of lactate [29]. Likewise, cancer-associated fibroblasts (CAFs) are known to release lactate into the extracellular space [30], further enriching the lactate pool that characterizes the tumour microenvironment.

## 3. Acidification of the Extracellular Medium

Lactate is exported from cells via the monocarboxylate transporters (MCTs) in symport with protons [31]. Consequently, the augmented release of lactate into the extracellular space results in a concomitant acidification of the extracellular medium [32] with in vivo measurements revealing extracellular pH values close to 6, as detected by hyperpolarized ^13^C MR imaging [33]; even at this low pH, the predominant form of lactic acid is in the anionic form lactate. This acidic microenvironment contributes to multiple cancer-promoting processes [34], including epithelial-to-mesenchymal transition, extracellular matrix degradation and remodelling, altered vascularization, and enhanced immune evasion [35]. Cancer cells adapt to such conditions through metabolic reprogramming mediated by PPARα signalling, which facilitates metastasis [36].

Acidic pH can act as a signalling cue, activating various acid-sensing ion channels and downstream pathways. These include increased reactive oxygen species (ROS) production and the activation of RhoA, NFAT, PI3K, and AKT-signalling cascades [37]. In addition, TME acidosis can trigger specific oncogenic signalling such as NF-κB activation, promoting cell invasion [38], and can drive immune escape through pathways involving SAT1 activation and interferon-γ–mediated upregulation of PD-L1 expression [39].

Although severe acidosis can trigger apoptosis [40], prolonged exposure to acidic pH allows tumoral cells to adapt and inhibit tumour necrosis factor-related apoptosis-inducing ligand (TRAIL)-induced apoptosis [41], thus facilitating tumour survival. However, lactic acid can induce apoptosis in resident NK cells [42], further facilitating tumour growth and dissemination. Also, lactate can supress autophagy [43] through lactylation [44].

High lactate concentrations and low extracellular pH play key roles in shaping interactions between tumour and stromal or immune cells within the tumour microenvironment [45]. Chronic acidosis creates a hostile niche for immune effectors [46,47]. Notably, Colegio and colleagues [48] demonstrated that lactic acid—but not lactate at physiological pH—induces a pro-tumoral macrophage phenotype, while acidity promotes tumour progression by reprogramming macrophages in prostate cancer [49]. Acidic pH also significantly attenuates the cytotoxic functions of T-cells [50,51]. Moreover, recent findings reveal that pH fluctuations can drive a reversible transition of fibroblasts into cancer-associated fibroblasts, further supporting tumour progression [52].

## 4. Lactic Acid as an Energy Source: The Reverse Warburg Effect

Lactate is a high-energy metabolite capable of yielding up to 32 molecules of ATP through mitochondrial OXPHOS when fully oxidized to CO_2_. Lactate generated by one tissue or cell type can be used by another for energy production, a process known as the *lactate shuttle*. The most well-known example is the Cori cycle, first described by Carl and Gerty Cori. This cycle describes lactate transfer from muscle to liver, where lactate is converted to glucose that can be returned to muscle to sustain activity when energy demand exceeds oxygen supply [4]. Although the Cori cycle has a net energy cost of −2 ATP molecules, it enables prolonged muscle function under hypoxic conditions. Lactate shuttling also occurs between different cell populations within a tissue; for example, astrocytes produce lactate that is released extracellularly and subsequently metabolized by mitochondria in neurons [53].

Lactate shuttles have also been observed within the tumour microenvironment, where this phenomenon is frequently referred to as the reverse Warburg effect. A tumour can be viewed as an evolving metabolic ecosystem in which cancer cells adopt strategies to optimize the use of available resources and regulate access to energy metabolites [54,55]. One form of metabolic symbiosis involves tumour cells in oxygenated regions metabolizing lactate secreted by hypoxic tumour cells that rely on glycolysis and cannot perform mitochondrial OXPHOS [56]. In breast cancer, tumour cells preferentially use lactate in oxygen-rich areas [57], further supporting the concept of intra-tumour lactate shuttling based on variable oxygen availability. Evidence from head and neck cancers shows the concurrent expression of MCT1 and MCT4 [58], further supporting the idea of metabolic symbiosis between lactate-producing and lactate-consuming tumour cell populations. 

The use of lactate as a fuel source appears to be widespread among cancers. Studies have shown that non-small-cell lung cancers (NSCLCs) can metabolize plasma lactate by incorporating it into the Krebs cycle (TCA cycle), a process that is dependent on the expression of MCT1 [59]. KRAS-mutant lung adenocarcinomas display greater glycolytic dependence than KRAS wild-type tumours, with LDHB expression correlating with poor survival [60]. LDHB, which catalyzes the conversion of lactate to pyruvate [24,61,62], is considered a key mediator of the reverse Warburg effect, suggesting that lactate utilization confers a metabolic advantage to tumours.

Metabolic symbiosis also occurs between cancer cells and stromal components. CAFs often exhibit elevated glycolysis and increased lactate export into the microenvironment [63]. The contact between CAFs and prostate cancer cells induces a mutual metabolic rewiring: stromal fibroblasts increase GLUT1 expression, lactate production, and export via MCT4, adopting a glycolytic phenotype. Conversely, cancer cells shift toward an aerobic OXPHOS metabolism, exhibiting decreased GLUT1 expression and increased lactate uptake via MCT1 [30]. CAF-derived lactate is metabolized via the Krebs cycle to meet the high energetic demands of tumour cells [64]. This process involves the activation of the SIRT1/PGC-1α axis, enhancing mitochondrial respiration in tumour cells and hijacking CAFs mitochondria through the formation of cellular bridges [65].

A stromal-epithelial lactate shuttle has also been demonstrated in breast cancer where co-culture of breast cancer cells and fibroblasts results in MCT4 upregulation in CAFs and MCT1 upregulation in MCF7 cells [59]. Similar patterns have been observed in human breast cancer samples, with CAFs from surgical resections showing higher MCT4 expression and lactate secretion compared to normal fibroblasts [66]. Furthermore, CAFs in hepatocellular carcinoma act as metabolic hubs, enhancing glycolysis and secreting lactic acid to the TME to fuel tumour growth [67]. While the existence of a lactate shuttle or reverse Warburg effect within the TME is widely accepted and clearly demonstrated in preclinical experimental settings, in humans, it is inferred from gene expression or protein localization experiments (see, for example, [55,68]). The lactate shuttle has been documented in human breast [69] and lung [59] cancer, among others, but the relative impact on tumour progression and response to therapy must be fully defined.

Beyond its metabolic role, CAF-derived lactate exerts direct immunosuppressive functions in pancreatic cancer TMEs [70]. They promote tumour invasion by releasing lactic acid, which induces M2 polarization in macrophages [68]. Also, CAFs excrete lactic acid through MCT4 that reduces apoptosis in tumoral cells, further supporting the idea that metabolic coupling between CAF and tumoral cells promotes aggressiveness [71]. Conversely, tumour-derived lactate can activate CAFs, stimulating IL-8 secretion that alters macrophage polarization dynamics [72]. Furthermore, lactate secreted by gastric cancer cells induced CAFs to express BDNF and promoted resistance to anlotinib [73].

## 5. Lactate as a Signalling Molecule

**Figure 1:**
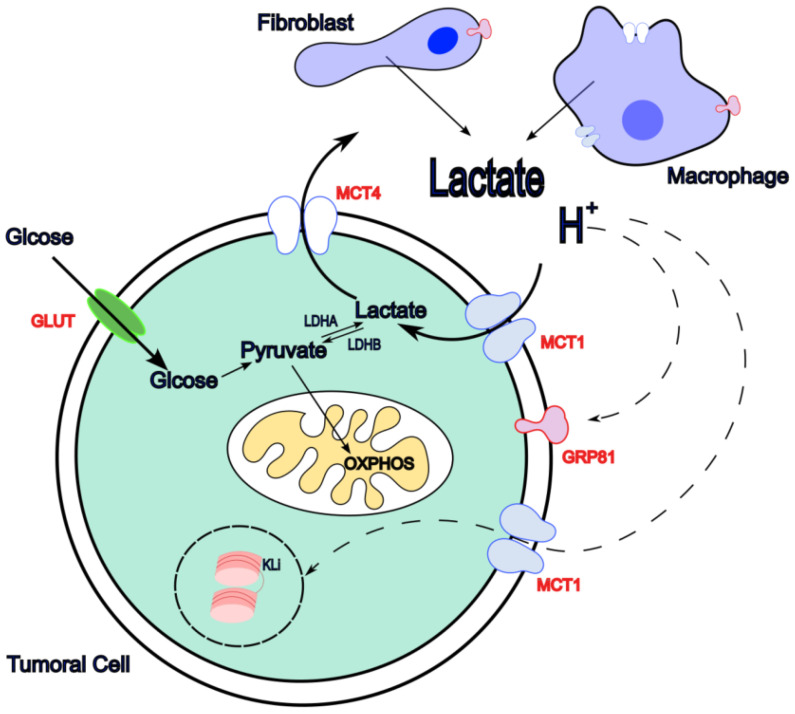
Metabolism of lactic acid in the TME (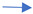). Glucose enters the cell via GLUT transporters, where it is metabolized to pyruvate and lactate that is exported from the cell through MCT4 transporters together with protons. This leads to the accumulation of lactate and acidification of the extracellular medium. Stroma cells can also contribute to TME lactate accumulation. Lactic acid can also enter the cell through the MCT1 transporter and can be incorporated into tumour cell OXPHOS metabolism, a process known as the reverse Warburg effect. Lactic acid can induce signalling (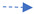), mainly through lactate receptor GPR81, but also through histone and other protein lactylation and MCT1 as a receptor. Lactic acid-induced signalling pathways can affect tumoral and stromal cells where they tend to induce a pro-tumoral phenotype in immune cells.

Within the tumour microenvironment, lactate functions not only as a metabolic by-product but also as a potent signalling molecule. It can interact with membrane-bound receptors, most notably the G protein-coupled receptor GPR81 (Figure 1), encoded by the *HCAR1* gene [74,75]. The primary agonist of GPR81 is lactic acid and its conjugate base lactate. GPR81 expression correlates positively with tumour growth and metastasis, and genetic deletion of GPR81 markedly suppresses tumour progression [76]. Activation of the GPR81 signalling axis induces a metabolic shift in cancer cells by enhancing glycolysis, creating an apparent autocrine feed-forward loop [77,78,79]. Notably, GPR81 expression is associated with the expression of primary lactate transporters MCT1 and MCT4 [80], supporting the concept of lactate-driven autocrine regulation.

GPR81 activation also contributes to the creation of an immunosuppressive microenvironment within the TME [81]. Lactate dampens a pro-inflammatory response in macrophages through GPR81 receptor signalling [82]. It also facilitates the recruitment of immunosuppressive polymorphonuclear myeloid-derived suppressor cells by upregulating CCL2 and CCL7 [83]. In antigen-presenting cells within the TME, GPR81-mediated signalling supports breast cancer growth via paracrine mechanisms [82,84]. Additionally, in gastric cancer, it promotes regulatory T-cell (Treg) migration through CX3CL1 secretion [85]. Furthermore, GPR81 has also been postulated as a key mediator in the induction of cancer-associated cachexia [86].

Lactic acid can further signal via the proton-sensing GPR132 receptor, which is highly expressed in macrophages and other hematopoietic cells but largely absent from most cancer cells, thereby contributing to macrophage reprogramming and metastasis [87].

Beyond classical receptors, MCT1 itself has been reported to initiate signalling cascades independent of ambient lactate [88]. Lactate can promote metastasis in normoxic colorectal cancer stem cells through PGC-1α-mediated signalling [89], indicating that lactate signalling is not confined to hypoxic tumour regions.

In addition to receptor-mediated signalling, lactic acid may play a role in the regulation of gene expression through *lactylation* (Figure 1)—a post-translational modification in which lactate-derived groups are added to lysine residues on histones [90]; this reshapes chromatin structure and alters gene expression, enzymatic activities, and cytokine production, among other effects. The extent of histone lactylation is directly proportional to intracellular lactate production, such as that driven by the Warburg effect, and has been shown to induce M2-like gene expression in macrophages [90]. This mechanism has since been found to extend to non-histone proteins [91]. For instance, the protein High Mobility Group Box-1 (HMGB1) was observed to undergo lactylation in macrophages during septic conditions [92]. Lactylation is a context-dependent mechanism [93], for example, when lactylation occurs in histones, it modifies gene expression such as in the case of M2 like polarization of macrophages, where it was observed that extracellular lactate accumulation altered M2 gene transcription and functional immune suppression though a process requiring functional mitochondrial metabolism [94]. On the other hand, when lactylation occurs in non-histone proteins, it can result in the activation of signalling pathways such as in Treg, where lactate induces lactylation of MOESIN; this in turn activates TFG-β signalling, which leads to a reduced anti-PD-1 treatment response [95].

Lactate within the TME can also induce lactylation in tumour cells; for example, CAF-derived lactate drives the lactylation of histone H3 at lysine 18 (H3K18) in gastric cancer, promoting immune evasion [96]. In acute lymphoblastic leukemia, sphingomyelin-induced lactic acid production led to caspase-3 lactylation, which was shown to subsequently inhibit apoptosis [97]. Additionally, lactylation can facilitate the progression of colorectal cancer by establishing a positive feedback loop that further enhances lactate production [98]. A comprehensive overview of lactylation-dependent regulation in the TME is provided in the review by Zhou and colleagues [99].

## 6. Effects of Lactic Acid on the Immune System

The tumour microenvironment promotes immune evasion through multiple mechanisms [100], including the secretion of immunosuppressive cytokines such as TGF-β, [101] and LIF [102]. The behaviour of immune cells within the TME is shaped by various local factors, among which lactic acid plays a critical role [103,104]. Lactate, one of the most abundant metabolites in tumours, functions as both a metabolic substrate and a signalling molecule, acting through several receptors [74] to influence immune responses across diverse conditions [90]. Metabolic reprogramming of immune cells drives immunosuppressive phenotypes within both innate [105] and adaptive immune populations [106], impacting macrophages, T-cells, myeloid-derived suppressor cells (MDSCs), and other stromal or immune-associated cells (Table 1).

### 6.1. Macrophages

Macrophages are integral drivers of cancer pathophysiology, influencing tumour initiation, progression, and metastasis [107]. Their phenotypes and functions are tightly regulated by metabolic cues within the TME [108,109]. As noted earlier, lactic acid promotes the polarization of tumour-associated macrophages (TAMs) toward an immunosuppressive (M2-like) phenotype, enhancing tumour growth and survival in murine models [23,48] and humans [23,48]. Importantly, it is exogenous lactic acid imported via MCT1 that drives this polarization process [110]. This shift in macrophage phenotype is accompanied by a reciprocal metabolic reprogramming within the macrophages themselves [108]. Studies utilizing microfluidic systems demonstrated that lactic acid induces M2-like polarization in macrophages more rapidly than larger, slower-diffusion, microenvironmental proteins [111].

**Figure 2:**
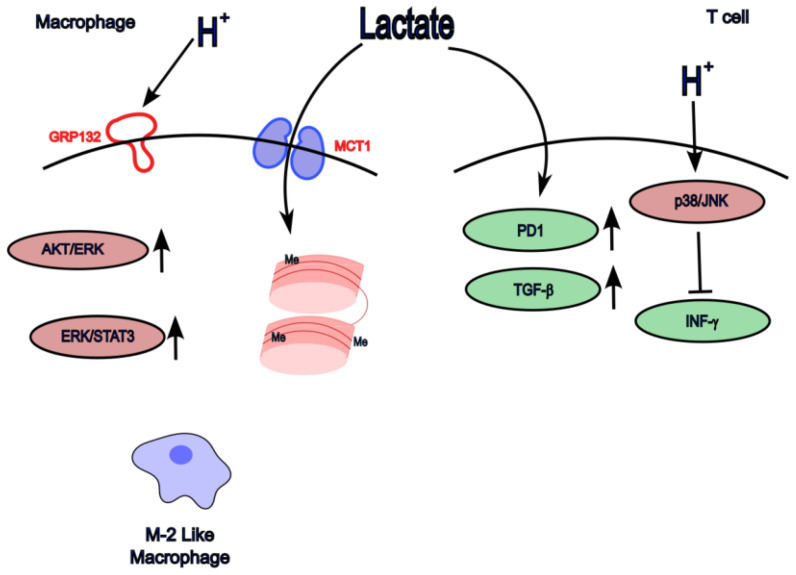
Effects of lactic acid on the immune system. Lactic acid can induce M-2-like macrophage polarization through various mechanisms including the activation of ERK/STAT3, AKT/ERK signalling pathways, or lactlylation. T-cell function can also be altered by lactic acid. Not all processes have been described in the same cell or tumour.

Lactate-induced macrophage polarization has been reported across multiple cancer types. In head and neck cancer, lactate-dependent M2 polarization correlates with tumour aggressiveness [23]; in a breast cancer model, lactate induces M2 polarization through ERK/STAT3 pathway activation [112], and in oesophageal cancer, lactate enhances tumour growth via AKT/ERK signalling [113]. However, in those reports, the authors did not disclose pH; thus, it is impossible to evaluate the effects of extracellular medium acidity. The lactate receptor GPR132 is abundant in hematopoietic-derived cells and contributes to macrophage-mediated immune regulation within the TME, particularly in lung adenocarcinoma [87,114]. In this case, the experimental design allowed for effects to be attributed directly to lactate and not acidic pH alone [87,114]. Moreover, lactate metabolism, through its conversion to pyruvate and subsequent mitochondrial oxidation—a process resembling the reverse Warburg effect—enhances histone acetylation and promotes the pro-tumoral macrophage phenotype [94,110] (Figure 2). Furthermore, in oral squamous cell carcinoma, tumour cell-derived lactic acid induces macrophage synthesis of glycoprotein non-metastatic protein B (GPNMB), which facilitates tumour cell migration and invasion [115]. Macrophages, in turn, interact with other immune cells, contributing to the suppression of T-cell recruitment [116].

### 6.2. T-Cells

Effector T-cells rely on glycolytic metabolism for their functions. Consequently, they are detrimentally affected in the TME due to the synergistic effects of low glucose availability and high lactate concentrations, which severely compromises their antitumour cytotoxic activity [19]. These changes were found to be specific to CD8+ T-cells isolated from tumours [117], suggesting that conditions in the TME might impact CD8+ T-cell metabolic reprogramming.

Lactate accumulation further compromises T-cell cytotoxicity and fosters the differentiation of regulatory T-cells (Tregs) into pro-tumoral phenotypes. Inhibition of MCT1 disrupts lactate uptake and reduces the Treg-mediated suppression of antitumour responses [118]. In NSCLC, an LTB^+^LDHA^+^CD8^+^ T-cell subset exhibits enhanced glycolysis and lactate production, promoting tumour cell migration while impairing CD8^+^ cytotoxicity [119]. Acidic pH suppresses CD8^+^ T-cell activity via p38/JNK pathway inhibition and reduces interferon-γ production (INFγ) [120]. Furthermore, lactic acid stimulates PD-1 expression in Tregs [121] and activates TGF-β signalling, reinforcing their immunosuppressive profile [95]. Interestingly, the transcriptional factor MondoA-induced thioredoxin interacting protein (TXNIP) is involved in the immunosuppressive function induced by lactic acid in both Treg and CD8+ cells [122].

### 6.3. Other Stromal and Immune Cells

The immunosuppressive composition of the TME also affects additional cell types. Lactic acid prevents monocyte differentiation into mature dendritic cells, promoting a tolerogenic phenotype and the secretion of anti-inflammatory cytokines [123]. Natural killer (NK) cell cytotoxicity is impaired under high lactate conditions through inhibition of the mTOR pathway [124]. Moreover, lactic acid can modulate NK cell activity via the proton-sensing receptor GPR132 [125].

## 7. Lactic Acid and Senescence

Cellular senescence is a stress-induced state characterized by permanent proliferative arrest and heterogeneous phenotypes that may exert both antitumour and tumour-promoting effects [126]. While senescence can suppress tumour growth by halting the proliferation of malignant cells, senescent cells often display enhanced resistance to apoptosis [127].

Metabolically, senescent cells retain mitochondrial activity but exhibit an increased glycolytic rate, resulting in elevated lactate production, which can contribute to tumour progression [128]. Lactate can modulate the senescence phenotype in lung cancer cell lines via Snail signalling [129], with a portion of this lactate originating from CAFs, thereby supporting tumour growth and metastasis [130]. Conversely, lactate may also enable certain hepatocellular carcinoma cells to bypass or resist senescence, further promoting malignancy [131].

**Table 1:** Main effects of lactic acid in the cellular compartments of a tumour.

Target	Effector	Result	Mechanism	Ref.
Tumoral Cells	Lactic acid	Tumour progression	GPR81	[76]
		Immune evasion	Lactlylation	[96]
		Inhibition of apoptosis	Lactlylation	[97]
		Cell migration and invasion	Lactlylation	[132]
		Senescence	SNAIL	[129]
		Senescence		[131]
	Low pH	Cell Invasion	NF-κB	[38]
		Increase in PD-L1	STAT1 INF-γ	[39]
Macrophages	Lactic Acid	Pro-tumoral phenotype		[23,48]
		Pro-tumoral phenotype	Lactlylation	[90,94]
		Pro-tumoral phenotype	ERK/STAT3	[113]
		Pro-tumoral phenotype	AKT/ERK	[99]
		Pro-tumoral phenotype	GPR132	[100,101]
T-cells	Low pH	Attenuates T-cell function		[43,44]
		Attenuates T-cell function	P38/JNK	[106]
	GPR81	T-cell migration	CX3CL1	[76]
	Lactic acid	T-cell apoptosis		[120]
	Lactic acid	Immunosuppressive function	PD-1 Expression	[121]
	Lactic acid	Immunosuppressive function	Lactylation, TGF-β	[95]
	Lactic acid	Immunosuppressive function	Mondo-A	[122]
NK Cells	Lactic acid	Impaired cytotoxicity	GPR132 mTOR	[125]
	Lactic acid	Impaired cytotoxicity		[124]
	Lactic acid	Apoptosis		[42]
Fibroblasts	Low pH	Transition to CAF		[52]

## 8. Effects Beyond the Microenvironment: Lactic Acid and Metastasis

Elevated lactate concentrations within the tumour microenvironment (TME) extend their influence beyond the primary tumour, driving metastatic dissemination [133]. Metastasis accounts for over 90% of cancer-related mortality and thus represents a major therapeutic challenge [134]. For metastasis to occur, cancer cells must undergo profound metabolic adaptations, with both lactate and pyruvate playing key roles in enhancing migratory and invasive capabilities [45].

In pancreatic cancer, increased lactate production—quantified by extracellular acidification rate (ECAR)—correlates positively with metastatic potential [135]. Conversely, highly metastatic melanomas display enhanced lactate uptake [136]. These seemingly paradoxical findings reflect lactate’s dual roles in tumour biology as both a product of glycolysis and an oxidative fuel. In breast cancer, activation of LDHA induces H3K18 lactylation and upregulates its own expression in a positive feedback loop, leading to increased lactate production, cell migration, and invasion [131]. In parallel, MCT1-dependent lactate transport and the reverse Warburg effect contribute to metastasis through lactylation of Rab7a [137].

As stated above, lactate also modulates the behaviour of stromal and immune components within the TME, thereby facilitating metastasis. It regulates T regulatory cell activity [95] and, in NSCLC patients, it has been shown that elevated LDHA expression is linked to increased lactate formation, CD8^+^ T-cell immune evasion, and enhanced metastatic progression [119]. Tumour-associated macrophages (TAMs) similarly drive tumour growth and metastatic spread [138,139]. Lactate secreted by cancer cells activates M2-like macrophages via GPR132 signalling, promoting migration and invasion through paracrine mechanisms [87]. Additionally, lactate induces PD-L1 expression in macrophages through activation of the NF-κB pathway, fostering formation of pre-metastatic niches [140].

Beyond immune modulation, lactate influences the extracellular matrix architecture, which is critical for invasion. In prostate cancer, lactic acid stimulates collagen I production, enhancing cellular invasiveness [141]. Similarly, intracellular conversion of lactate to pyruvate promotes collagen deposition, which sustains cancer stem cell populations essential for metastatic outgrowth [142].

## 9. Clinical Implications

### 9.1. Therapeutic Targeting of Lactate Metabolism

As reviewed above, lactate exerts multiple deleterious effects on tumour biology by promoting invasiveness, metastasis, and immune evasion. Consequently, therapeutic strategies aimed at limiting lactate accumulation within the tumour microenvironment have emerged as promising anticancer approaches [143].

One potential target is LDH [144], the key enzyme responsible for lactate synthesis. Inhibition of LDH has been shown to impede tumour progression [145], suggesting that interfering with lactate production may suppress tumour growth. However, subsequent studies proposed that the therapeutic benefit of LDH inhibition arises partly from the modulation of immune activity through IL-21 signalling [146]. Alternatively, it has been shown that the inhibition of LDH increases glucose availability in the TME leading to improved tumour-killing T-cell function and impaired Treg immunosuppressive activity in vitro. In a murine model, the combination of LDH inhibition with immune checkpoint blockade therapy effectively controlled melanoma and colon cancer progression by promoting effector T-cell infiltration and activation, while destabilizing Tregs [147]. While these studies noted reductions in intratumoral lactate, the specific contribution of lactate depletion to these effects has yet to be conclusively determined.

An alternative strategy focuses on disrupting lactate transport. Pharmacological inhibition of lactate export through MCTs showed preclinical promise. The selective MCT1 inhibitor AZD3965 was investigated in a phase I clinical trial, where it demonstrated good tolerability at doses sufficient to block lactate transport [148,149]. Further studies are needed to refine biomarkers for patient selection. Additionally, VB124—a recently developed MCT4-specific inhibitor—has shown efficacy in animal models [150,151]. Preclinical data has shown that pharmacological lactate reduction in the TME also enhances immunotherapy [122,146,152]. Randomized clinical trials are needed to evaluate lactate-lowering strategies combined with immunotherapy and other anticancer agents in clinical practice.

### 9.2. Imaging and Biomarker Applications

Communication between the TME and the peripheral circulation may also allow tumour-derived metabolites to serve as minimally invasive biomarkers [153]. Although systemic lactate levels are tightly regulated [79], elevated plasma lactate could reflect tumour metabolic activity. Metabolomic profiling has revealed higher serum lactate levels in patients with non–small-cell lung cancer compared to healthy controls, with concentrations increasing alongside disease severity [154]. In multiple myeloma, serum lactate predicted poor clinical outcomes and correlated with decreased Bortezomib sensitivity by a reduction in apoptosis [155]. Retrospective analyses further support lactate’s prognostic value: in a study of 85 patients with stage IV NSCLC or small-cell lung cancer, plasma lactate correlated with metastatic burden even after adjusting for confounders such as intubation and ICU admission [156]. Elevated plasma lactate was also associated with reduced overall survival in lung cancer [156]. Similar findings have been reported in ovarian cancer [157], lymphoma [158], prostate cancer [128], and colorectal cancer, where lactate levels were significantly higher in metastatic versus non-metastatic patients [159]. In the case of breast cancer, lactate levels measured in whole tumour freshly excised samples correlated with tumour grade and Nottingham Prognostic Index, but not with proliferation rate as measured by Ki67 expression [160]. On the other hand, a high correlation between Ki67 and lactate levels was found in gliomas [161]. Interestingly, lactate concentrations decline in multiple myeloma patients responding to therapy [162], suggesting that serum lactate could serve as a dynamic marker for treatment efficacy.

Given that lactic acid accumulation is a common feature across diverse tumour types, its potential as a diagnostic and prognostic biomarker has been the focus of growing interest. Using ^13^C-hyperpolarization magnetic resonance spectroscopy, changes in TME lactate production can be detected within 24 h after drug administration, often preceding conventional radiological indicators of therapeutic response [163].

## 10. Challenges and Open Questions

Preclinical studies have clearly demonstrated that lactic acid plays a central role in tumour development and response to treatment (Table 2). However, not all mechanistic studies are properly designed to differentiate between the effects of lactic acid, the ion lactate, or extracellular acidification. While some published works deserve praise for considering the possible differences between lactic acid, lactate, or acidic pH (see for example: [48,87,94,95]), others just state that the effects observed are due to “lactic acid” (see for example: [113,114,132]). Future work must rigorously dissect whether lactate, low pH, or their combination drives observed phenotypes.

Lactic acid apparently contributes to malignancy in a widespread manner, but it is unclear whether its specific role and weight vary among different tumour types and stages. Since lactic acid occurs independently of tumour type, the idea of using lactate as a therapeutic target or biomarker is tantalizing. Targeting lactate metabolism as monotherapy is challenging because it has a central role in metabolism and it is not cancer-specific [8]; however, reducing TME lactate levels may enhance immunotherapy response [95,122].

Circulating lactate monitoring could serve as a pan-cancer biomarker for tumour progression and therapy response. However, factors other than cancer, for example, shock and sepsis [19], can increase blood lactic acid concentration and should be controlled to avoid interferences. Incorporating lactate, along with key enzymes (LDH) and transporters (MCTs), into prospective biomarker panels would clarify its utility.

Current understanding of lactate’s role in tumour biology relies heavily on preclinical data, with limited in vivo human evidence. The lack of prospective clinical trials prevents full evaluation of lactic acid as a biomarker; however, several retrospective studies are promising, albeit with all the caveats associated with retrospective studies such as patient selection bias. In this context, key open questions remain to be clarified and pose a significant challenge.

The serum metabolic profile differs between males and females [165], and in the case of glioma patients, differences in expression of genes related to glycolysis have been reported [166]. A higher glycolytic rate was also found in right colon samples from men as compared to women [167]. A recent study showed that melanoma samples from male patients had increased expression of LDHA, HKII, and MCT4 and were able to produce more lactate ex vivo than samples from female patients; the increase in glycolytic function was accompanied by a reduction in functional CD8+ T-cells [168]. Tumour metabolic differences associated with the patient’s sex might affect treatment response [168]; thus, this should also be considered when designing experiments and clinical trials and analyzing the data.

Translating preclinical lactate findings to patients remains challenging due to difficulties measuring true TME lactate concentrations in vivo. Nevertheless, imaging studies suggest that lactic acid production can be monitored non-invasively to assess treatment response [165], warranting further clinical exploration.

## 11. Concluding Remarks

Once regarded merely as a metabolic waste product, lactate is now recognized as a central player in cancer biology. Tumour cells can exploit lactate as an efficient energy source via mitochondrial oxidative phosphorylation (OXPHOS) and can induce stromal cells to enhance its production, fostering a metabolically supportive microenvironment. Beyond its metabolic role, lactic acid functions as a signalling molecule—acting through receptors such as GPR81 or via direct protein lactylation—to drive immunosuppression, promote metastasis, and modulate diverse aspects of tumour progression.

Therapeutic strategies aimed at reducing lactate abundance or its downstream effects in the TME are gaining traction. Approaches include inhibiting lactate production, disrupting its transport, or targeting lactate-mediated signalling pathways. In parallel, lactate is being investigated as a prognostic biomarker and a potential indicator of treatment response, offering both mechanistic insights and clinical utility in oncology.

## Figures and Tables

**Table 2:** Key Milestone findings in chronological order.

Authors	Year	Key Findings	Ref.
Warburg	1956	Demonstration that tumoral cells produce lactate.	[164]
Walenta et al.	2000	Lactic acid as a biomarker. Correlation between tumour lactate concentration in biopsies with patient survival.	[9]
Day et al.	2007	In vivo tumour lactate production is an early marker of therapeutic response.	[164]
Fischer et al.	2007	Lactic acid inhibits T-cell function.	[50]
Gallager et al.	2008	In vivo TME pH values close to 6 in animal models.	[33]
Whitaker-Menezes et al.	2011	Evidence of a lactate shuttle between tumour and stroma (CAF) cells.	[66]
Capparelli	2012	Lactic acid can modulate senescence.	[130]
Colegio et al.	2014	Lactic acid, but not lactate at neutral pH, is able to polarize macrophages towards a pro-tumoral phenotype.	[48]
Vlachosterios et al.	2015	Retrospective clinical study showing that serum lactic acid is a negative prognostic factor in lung cancer.	[156]
Zhang et al.	2019	First evidence of lactate-induced post-transcriptional modification of histones; lactylation.	[90]

## Data Availability

No new data were created or analyzed in this study.

## Abbreviations

The following abbreviations are used in this manuscript:

MondoA-induced thioredoxin interacting protein

TME	Tumour microenvironment
LDH	Lactate dehydrogenase (A or B)
MCT	Monocarboxylate transporter (1 or 4)
OXPHOS	Oxidative phosphorylation
CAF	Cancer-associated fibroblast
TAM	Tumour-associated macrophage
LIF	Leukemia inhibitory factor
NSCLC	Non-small-cell lung cancer
TXNIP	MondoA-induced thioredoxin interacting protein
TRAIL	Tumour necrosis factor-related apoptosis-inducing ligand
